# Investigating the Factors Influencing Traditional Male Circumcision and Its Contribution to HIV Transmission Amongst Men in Lesotho: A Multilevel Binary Logistic Regression Approach

**DOI:** 10.3390/ijerph22070993

**Published:** 2025-06-24

**Authors:** Sizwe Vincent Mbona, Anisha Ananth, Retius Chifurira

**Affiliations:** 1 Department of Statistics, Faculty of Applied Sciences, Durban University of Technology, Durban 4001, South Africa; anishas@dut.ac.za; 2 School of Mathematics, Statistics and Computer Science, University of KwaZulu-Natal, Durban 4001, South Africa; chifurira@ukzn.ac.za

**Keywords:** culture, Lesotho, multilevel logistic regression, TMC

## Abstract

Background: Traditional Male Circumcision (TMC) has been practiced in many parts of the world. However, the impact thereof on reducing HIV transmission is still unclear. This study aimed to examine the prevalence and determinants of TMC and the associated risk of HIV transmission in Lesotho. Method: Using data from the 2023–24 Lesotho Demographic and Health Survey, the analysis included a weighted sample of 3202 men aged 15–59 years. Missing data was addressed through multiple imputations, and multilevel logistic regression was used to assess the factors associated with TMC, incorporating intra-class correlation to evaluate cluster-level variation. Results: The findings revealed that 51.0% (95% CI: 49.3–52.7) of men in Lesotho had undergone TMC. Older men, particularly those aged 35 years and above, were more likely to be circumcised compared to younger men aged 15–24 years (AOR = 1.63; 95% CI: 1.46–1.86). Other individual-level factors positively associated with TMC included smoking, being married to one partner, previous sexual experience, and rural residence. Conversely, men with formal education, unknown or undisclosed HIV status, residing in the Berea or Maseru districts, and those from middle- or high-income households were less likely to undergo TMC. Conclusion: The study highlights significant variation in TMC practices across communities and identifies both individual and contextual factors influencing its uptake. These insights underscore the need for culturally sensitive, voluntary, and medically safe circumcision programs. Public health initiatives should consider these determinants when designing interventions to ensure a safer and more effective implementation of TMC in Lesotho.

## 1. Introduction

Traditional Male Circumcision (TMC) is the practice that entails the cutting the foreskin of the penis on young men for cultural and religious beliefs [1]. TMC is normally performed non-clinically by the traditional headman who has never attended any formal medical training, and who is only chosen by the society [2]. The ritual is not discussed with outsiders, including females and males who have not undergone it [3]. TMC has been practiced in many countries in the global North and South, namely Australia, Asia, Africa, Polynesia, South and North America [4]. After undergoing TMC, the initiate is required to shout ‘I am now a man’, which proves manhood and is also a way of trying to ignore the pain [5]. This differentiates a so-called ‘bush man’ from a ‘hospital man’, because when male circumcision (MC) is performed in hospitals, the individual is given painkillers and there is no need to test the initiate [6]. About 86% of individuals who undergo traditional circumcision experience intense pain. In some cases, serious long-term complications may arise, such as erectile dysfunction, chronic swelling, heavy scarring, and even partial or complete penile amputation. These severe outcomes are responsible for approximately 14% of hospital admissions related to TMC [7].

Notwithstanding the potential risks that undergoing TMC poses, it is considered a vital public health measure, as MC carries significant protection against human immunodeficiency virus (HIV) transmission and other sexually transmitted infections [8]. Consequently, MC is one of the strategies recommended by government and public health practitioners to reduce HIV transmission in places with a high HIV prevalence [9].

Although TMC has not yet been approved by the World Health Organisation (WHO) as a method of reducing HIV, many cultures are encouraging TMC practices amongst adolescent males as a preventive intervention against the spread of diseases, including HIV/AIDS and other sexually transmitted infections (STIs) [10,11,12,13]. However, findings from earlier studies suggest that TMC typically involves the removal of only a small portion of the foreskin, which may offer limited effectiveness in reducing the risk of HIV transmission [14]. Furthermore, males who are circumcised traditionally and have sexual intercourse before the wound completely heals are considered to have minimal protection [15]. However, the 2009 Demographic and Health Survey (DHS) in Lesotho found no noticeable variations in sexual risk behaviors amongst men who underwent medical male circumcision and those who had TMC [16]. A study conducted in Maputo City, Mozambique, found that men with no formal education or only primary education were three to four times more likely not to undergo circumcision. Not being circumcised was associated with lower rates of practicing safer sex and a higher occurrence of STIs, underscoring the need to strengthen comprehensive HIV prevention efforts and support regular screening for HIV and STIs [17].

Demographic factors such as age, income, education level, and relationship status were found to have a significant relationship with the willingness to undergo TMC. Although many researchers have conducted studies on MC, there is still no clear evidence of its positive impact on HIV transmission and STI prevention, and the topic remains a subject of debate amongst scholars [18]. A study found that diseases are frequently transmitted during the TMC ceremony because traditional surgeons share the circumcising sword, traditional nurses re-use bandages, and certain teachings promote risky sexual behaviors [19]. Some initiation schools teach ideas that support male dominance and the equitable treatment of women as less important. These taught value systems potentially increase the prevalence of gender-based violence [20]. This is emphasized by the findings of a South African study which exposed that TMC participants are subjected to negative teachings in initiation schools, which do not align with societal moral and ethical standards. These include the prevalence of violence and alcohol abuse. The study also highlighted a lack of adherence to moral codes and cultural values by contemporary TMC initiatives [21].

This research aimed to measure the variation in TMC across different sample enumeration areas or clusters; examine the impact of cluster characteristics on TMC using the intra-class correlation coefficient; and investigate the factors affecting TMC in Lesotho through a multilevel binary logistic regression approach. Many large-scale surveys like the DHS, epidemiological research, and public health research are based on multistage stratified cluster sampling, which often follows a hierarchical data structure. Since the 2023–24 LDHS employed a multistage stratified cluster sampling design and the outcome variable in this study is binary, appropriate statistical methods accounting for the complex survey design were applied. The researchers of this study applied a multilevel binary logistic regression method to analyze data to account for the clustering of individuals within clusters of higher-level units when estimating the effect of individuals and clusters. This approach is appropriate for such data because it involves variability nested within various levels of the hierarchy. It is essential to identify and understand the factors that influence TMC and how TMC contributes to reducing HIV spread. This knowledge helps healthcare workers and the government to create better, targeted solutions. In addition, it is important to understand how TMC affects men, their families, and communities.

## 2. Methods and Materials

### 2.1. Source of Data, Study Area, and Study Design

The data used in this study were drawn from the 2023–24 LDHS. Lesotho is in the southern region of Africa and is one of the landlocked countries of the continent. The 2023–24 LDHS sampling frame is derived from the 2016 Lesotho Population and Housing Census, which was carried out by the Lesotho Bureau of Statistics. The sampling frame provides a full listing of every census enumeration area (EA) across the country. An EA refers to a geographical unit, typically a city block in urban areas or a village in rural areas, containing roughly 100 households. This study utilizes data from the DHS due to its value as a nationally and regionally representative source that assesses a wide range of health and socio-economic indicators. The data are publicly accessible online upon request at https://www.dhsprogram.com/data/dataset_admin (accessed on 26 November 2024). The LDHS employed a complex survey design, which involved multistage, stratified, and cluster sampling with unequal probabilities of selection.

The number of predictors included in the study is 17, with one outcome variable which is binary. The study considered two TC outcomes for respondents (1 is interpreted as “traditionally circumcised” and 0 is interpreted as “not traditionally circumcised”). Independent variables used in this study were examined across two levels (individual and contextual levels), presented in Table 1. The variables categorized under the individual level included age, level of education, literacy, smoking status, coverage by health insurance, marital status, working status, HIV test result, whether the respondent would marry a person with HIV, number of wives/partners, self-reported health status, partner currently being pregnant, and whether they had ever had sex. The contextual-level variables included were place of residence, district, sex of household head, and wealth status [22]. Greater details and descriptions of these variables have been highlighted in the literature [23,24,25].

**Figure 1:**
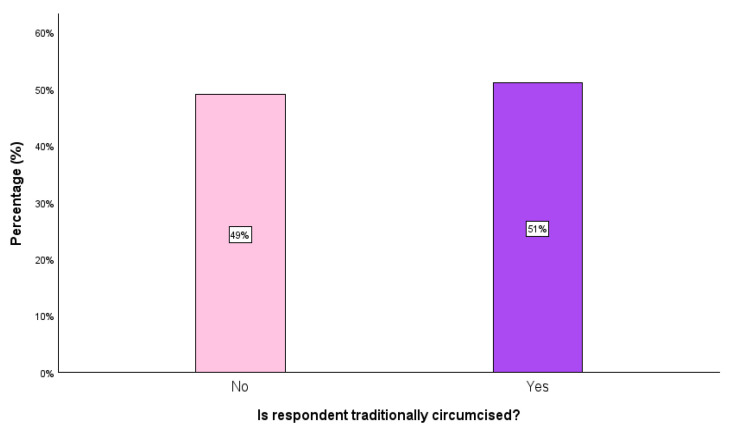
Target variable distribution.

The study utilized data collected from 3202 Lesotho men aged 15–59 years, with 49.0% being not traditionally circumcised and 51.0% being traditionally circumcised (Figure 1). The large sample size used in this study, which generally represents the target population, was designed to produce statistically valid estimates. Furthermore, the study applied a weighting variable to account for the complex survey design, ensuring both representativeness and accuracy [26]. This cross-sectional study was used to investigate important factors influencing TMC in Lesotho.

The researchers of this study grouped the age of men into 3 categories: 15–24, 25–34, and 35+ years [27]. Wealth status was simplified from five categories into three categories: ‘poorest’ and ‘poorer’ were combined into ‘poor’; ‘middle’ remained as ‘middle’; and ‘richer’ and ‘richest’ were grouped as ‘rich’. Similarly, self-reported health status was simplified from five levels to three levels, with ‘very good’ and ‘good’ combined as ‘good’; ‘moderate’ retained as ‘moderate’; and ‘bad’ and ‘very bad’ merged into ‘bad’ [28,29,30,31]. These variables were chosen based on their theoretical significance and their availability within the dataset.

### 2.2. Missing Data Management and Multicollinearity Assessment

**Figure 2:**
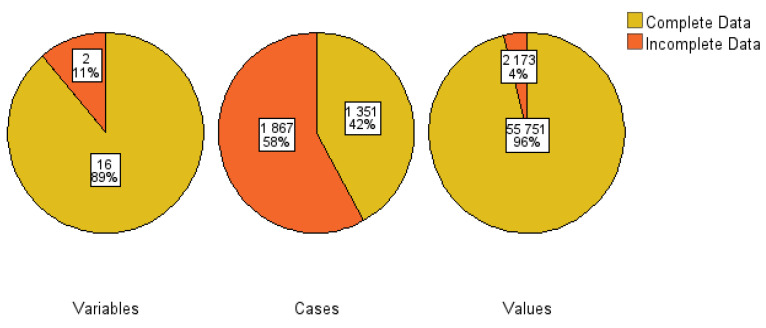
Overall summary of missing values.

Some of the factors examined in this study had missing values. The researchers first dealt with them by applying a method called multiple imputations by chained equations (MICE). This method is helpful in addressing the problem of missing values in datasets. The method is well presented by other studies in the extant literature [32,33]. In this study, missing values were treated as missing at random (MAR), hence the MICE method was employed. The *mice* package within R software was implemented for imputing missing values. Figure 2 shows a summary outlining the missing values in the dataset. The researchers observed that only two (11%) explanatory variables had incomplete data, 58% cases were missing, and 4% values in the entire dataset were missing.

The researchers employed the Variance Inflation Factor (VIF) to further evaluate multicollinearity, which indicated no significant multicollinearity amongst the variables (VIF values ranged from 1.52 to 3.46, with a mean VIF of 2.01). As a result, there was no need to drop or combine any highly correlated variables.

### 2.3. Model Formulation

The rate of TMC in Lesotho may vary across different regions due to environmental factors, regulations set by traditional chiefs, or other area characteristics. These variations can be incorporated into the model using random effects. Men residing in the same sample enumeration area (SEA) or cluster may exhibit more similarities to each other than to men living in different SEAs as they share common environmental factors and characteristics that could similarly influence TMC. This results in intra-class correlation, which assesses the level of similarity in TMC amongst men within the same cluster, such as an SEA. As a result, this study utilized a multilevel binary logistic regression model that incorporates SEA-specific random effects to address intra-class correlation and measure the variation in the TMC outcome explained by the differences between SEAs.

Suppose the outcome variable $\;y_{ij}$ of the i_th_ individual in the j_th_ SEA or cluster has two possible values: 1 if the man practiced TMC, and 0 if he did not. Let the probability of $y_{ij}$ be equal to $\pi_{ij}$. A logistic regression model with multilevel random effects for the outcome $y_{ij}$ is given by:

$$\log\left(\frac{\pi_{ij}}{1-\pi_{ij}}\right)=\beta {X\prime }_{ij}+\alpha {Z\prime }_{j}+b_{j},i=1,2,\ldots ,n\;and\;j=1,2,\ldots ,m$$

In the model, the $X_{ij}=\left(1,\;x_{1ij},\ldots ,x_{pij}\right)$ is the vector of p  predictors measured on the $\;i^{th}\;$ individual and $Z_{j}=\left(z_{1j},\;z_{2j},\;\ldots ,\;z_{pj}\right)$ is the vector of predictors measured on the j SEA (cluster). β and α are vectors of the fixed regression coefficient. The term $e^{\beta }$ is the odds ratio of TMC for X=x+1 compared to X=x. The term $b_{j}$ is the random effect varying over SEAs that has a normal distribution with zero mean and ${\sigma_{b}}^{2}$, in short $b_{j}\sim N\left(0,{\sigma_{b}}^{2}\right)$.

The variability between clusters in the multilevel logistic model was assessed using random effect measures such as the intra-cluster correlation coefficient (ICC) or variance partition coefficient, the percentage change in variance (PCV), and the median odds ratio (MOR). These indicators help explain how much the effects vary across different clusters [34]. The ICC, PCV, and MOR are calculated as follows:

$$ICC=\frac{\sigma^{2}}{\sigma^{2}+\frac{\pi^{2}}{3}}$$

where $\sigma^{2}$ is the variance between clusters (random intercept variance) and $\frac{\pi^{2}}{3}\approx 3.29$ is the level 1 residual variance.

$$PCV=\frac{{\sigma^{2}}_{null}-{\sigma^{2}}_{model}}{{\sigma^{2}}_{null}}\times 100$$

where ${\sigma^{2}}_{null}$ = between-cluster variance in the null model and ${\sigma^{2}}_{model}$ = between-cluster variance in the full model.

$$MOR=exp\left(\sqrt{2\sigma^{2}}\times 0.6745\right)$$

where 0.6745 is the 75th percentile of the standard normal distribution used to calculate MOR.

### 2.4. Statistical Analysis

Data were downloaded and edited in the Statistical Package for the Social Sciences (SPSS) version 29.20 and imported to R statistical software (version 4.4.2) for further analysis. The researchers presented the proportion of the men who performed traditional circumcision together with the distribution across all independent variables using percentages and a 95% confidence interval. In addition, the researchers conducted bivariate analyses to examine associations between the outcome variable and independent variables. The researchers applied a chi-square test of independence for comparing two categorical variables, whilst an independent sample *t*-test was applied for a continuous variable with the outcome variable. Furthermore, a multilevel binary logistic regression was employed by the researchers to examine the determinants of TMC. Individuals at a lower level were grouped across higher-level community clusters. To capture unexplained variation at the community level, clusters were included as random effects. The intra-class correlation coefficient (ICC) was computed. The ICC represents the proportion of variability accounted for by the clustering effect. To estimate the unadjusted (crude) odds ratios (COR), a bivariable multilevel logistic regression analysis was performed and independent variables found to be statistically significant were included in the adjusted multivariable multilevel logistic regression model [35]. Four multivariable multilevel logistic regression models were constructed to estimate adjusted odds ratios (AORs), accounting for confounding factors and assessing the degree of random variation between clusters:

Model 1 (the null model) did not include independent variables. Variation in TMC due to clustering at the primary sampling unit level is estimated by the fitted model and justifies the application of a multilevel analysis by computing ICC [36]. The null model was used as a reference point for comparing the other models. Model 2 included variables at the individual level to assess the impact of individual-level variables on TMC. Model 3 included variables at the contextual level to assess their impact on the outcome variable (TMC). Model 4 (the final model) included some variables from both Models 2 and 3 simultaneously. The model included only variables that were statistically significant in Models 2 and 3.

The findings were presented in terms of AORs and their respective 95% confidence intervals. The researchers interpreted the results from the complete model (Model 4) and used them in the discussion. Data used in this study were weighed in all analyses to account for non-responses and disproportionate samples as per DHS guidelines [37]. A significance level of *p* < 0.05 was used for the analysis.

### 2.5. Ethics Consideration

Authorization to access 2023 LDHS data was granted to the authors, and ethical approval and consent from participants was deemed unnecessary since the study used secondary data. Furthermore, all DHSs receive approval from the Inner-City Fund (ICF) International and the Institutional Review Board (IRB) in the host country to ensure compliance with the U.S. Department of Health and Human Services regulations for the protection of human subjects. The data is publicly accessible upon request and can be downloaded from http://www.dhsprogram.com/data/dataset_admin/login_main.cfm (accessed on 26 November 2024)

## 3. Result

### 3.1. Sociodemographic Characteristics of the Study Participants

Results displayed in Table 2 showed that the mean (±SD) age of men who participated in the study was 32.4 (±12.3) years. About 42.3% of the men studied were above 34 years old, followed by those between 15 and 24 years (35.0%) and 25 and 34 years (22.8%). Regarding educational level, 201 (6.3%) had no formal education; 1192 (37.2%) had a primary level of education; 1375 (42.9%) had a secondary level of education; and 434 (13.6%) had higher levels of education. The majority (74.5%) of men participating in the study were able to read a whole sentence, and 82.8% of them were covered by health insurance. More than half (54.8%) were non-smokers. With respect to marital status, nearly half (47.3%) of the men had never been in a union (single) and 42.3% were married. Furthermore, about 62.1% were working. The researchers also observed that 2486 (77.6%) men reported negative HIV results and only 15.8% indicated that they were HIV-positive. About 52.4% of the men indicated that they would marry someone who was HIV-positive. The results also reveal that 55.4% of the men had no wives/partners and 44.6% had only one wife/partner.

Regarding self-reported health status, 52.2% reported good health; 36.2% reported moderate health; and 11.6% reported bad health status. The study also observed that most (64.6%) of the men’s partners were not pregnant. Only 7.9% of the men reported never having had sex. More than half (58.8%) of the men were from rural areas and 41.2% were from urban areas. Most of the men (31.6%) were from the Maseru district (Table 2). The majority (74.3%) of households where these men came from were headed by males and 25.7% of the households were headed by females. About 1127 (35.2%) of the men were from poor households while 1370 (42.8%) were from rich households.

### 3.2. The Prevalence of Traditional Circumcision (TC)

The prevalence of TC in men from Lesotho was 51.0% (95% CI: 49.3–52.7). Traditional circumcision was highest amongst men aged 25–34 years (56.1%) and 35 years and above (54.8%). Furthermore, TC was more commonly reported amongst men with no formal education (87.6%), who could not read at all (82.1%), smoked cigarettes (64.6%), were covered by health insurance (53.4%), were married (56.6%), were not working (53.5%), were HIV-positive (58.4%), and had one wife/partner (56.5%). The researchers further observed that TC was reported more amongst men with a bad self-reported health status (63.0%), who had ever had sex (54.2%), resided in rural areas (62.4%), and those who came from households whose wealth index was reported as poor (75.2%).

**Figure 3:**
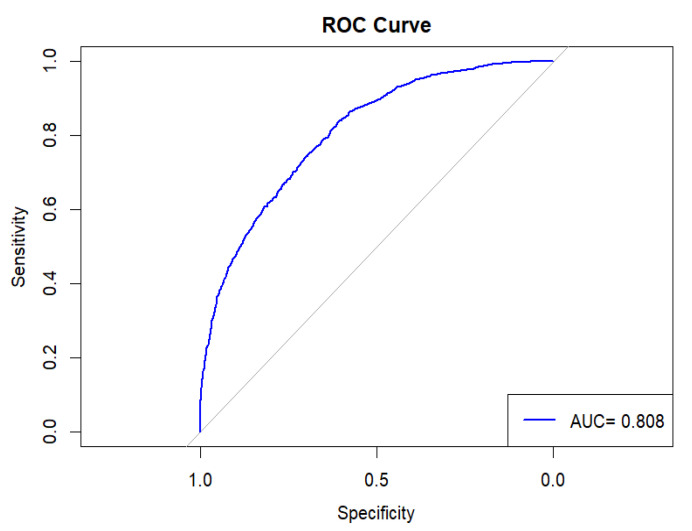
The Receiver Operating Characteristic (ROC) curve.

**Figure 4:**
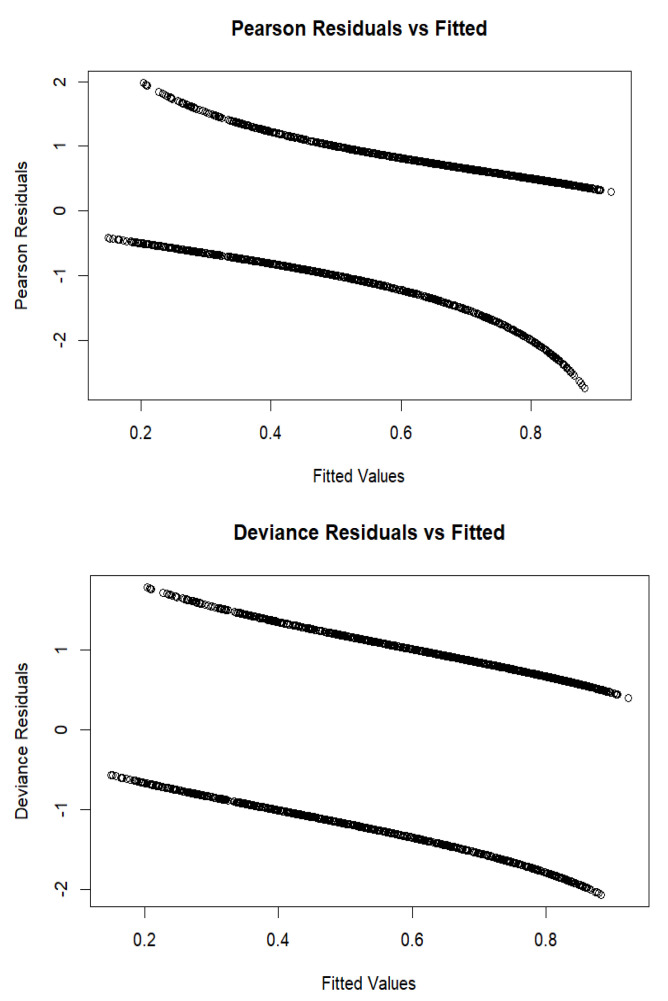
The Pearson and deviance residual plots against the fitted values.

The model’s performance was assessed using the Receiver Operating Characteristic (ROC) curve and the Area Under the Curve (AUC). The ROC curve shown in Figure 3 lies significantly above the diagonal reference line, demonstrating the model’s strong discriminative ability. This is supported by the AUC value of 0.808, which suggests that the model has an 80.8% probability of correctly distinguishing between positive and negative outcomes. In addition, the researchers plotted both Pearson and deviance residuals against the fitted values. As shown in Figure 4, most residuals fall within the range of −2 to +2, indicating a reasonably good model fit. This distribution suggests that the model’s predicted values align well with the observed data, with minimal deviations.

Table 3 displays the findings from both the univariable and multivariable multilevel logistic regression analyses. The findings indicate that the intra-class correlation in the baseline model (null model) was 0.29, suggesting that approximately 29% of the overall variance in TMC can be explained by variations in community-level factors (clusters). As the median odds ratio (MOR) in the full model (Model 4) was 1.69, this suggests that if men are randomly chosen from two different clusters, those from the cluster with higher odds are 1.69 times more likely to undergo TC in comparison to men from a cluster with lower odds of undergoing TC. The findings show that Model 4 performed the best, with the highest log-likelihood of −1986.202 and the lowest AIC value of 3804.416. The proportional change in variance (PCV) for Model 4 was 21.51, meaning that approximately 21.5% of the overall variation in TMC was accounted for by the complete model.

The multilevel logistic regression analysis in the final model revealed that individual-level factors, namely age (35+ years), education level, literacy, smoking status, marital status (married), HIV test results, number of wives/partners, and sexual history, were all significantly linked to TMC. On the other hand, community-level factors such as residence area, district, and wealth status were also significantly associated with TMC.

Considering other individual- and community-level factors, the results showed that men aged 35 years and older were 1.63 times more likely to undergo TC compared to men aged 15–24 years (AOR = 1.63; 95% CI: 1.46–1.86). Furthermore, men with primary, secondary, and higher education levels were 0.54 (AOR = 0.54; 95% CI: 0.30–0.97), 0.17 (AOR = 0.17; 95% CI: 0.09–0.32), and 0.06 (AOR = 0.06; 95% CI: 0.03–0.12) times less likely to undergo TC, respectively, when compared to men with no formal education. Men who could only read part of a sentence had a 41% lower chance of undergoing TC compared to those who could not read at all (AOR = 0.59; 95% CI: 0.37–0.93). Meanwhile, men who smoked cigarettes had 1.82 times higher odds of undergoing TC compared to those who did not smoke (AOR = 1.82; 95% CI: 1.50–2.21). The likelihood of undergoing TC amongst married men was about 1.43 times more common compared to those who were never in a union (AOR = 1.43; 95% CI: 1.07–1.91).

Men with inconclusive HIV test results and those who chose not to reveal their HIV status were 50% (AOR = 0.50; 95% CI: 0.27–0.93) and 74% (AOR = 0.26; 95% CI: 0.14–0.46) less likely to undergo TC, respectively, compared to men who tested HIV-negative. The likelihood of undergoing TC was 0.59 times lower amongst men who responded with “don′t know/not sure/depends” to the question “Would you marry someone with HIV?” compared to those who stated that they would never marry someone with HIV (AOR = 0.59; 95% CI: 0.37–0.95). Additionally, men with one wife or partner were 3.12 times more likely to undergo TC (AOR = 3.12; 95% CI: 1.71–5.13) compared to those with no wives or partners. Men who had ever engaged in sexual activity were 12.42 times more likely to undergo TC compared to those who had never had sex (AOR = 12.42; 95% CI: 7.69–20.07).

Men living in rural areas were 1.54 times more likely to undergo TC (AOR = 1.54; 95% CI: 1.12–2.12) than those living in urban settings. Compared to men from the Butha-Buthe district, those from the Berea and Maseru districts had 61% (AOR = 0.39; 95% CI: 0.22–0.70) and 67% (AOR = 0.33; 95% CI: 0.19–0.57) lower odds of undergoing TC, respectively. In terms of household wealth, men from middle-income households were 28% less likely (AOR = 0.72; 95% CI: 0.55–0.95) and those from wealthier households were 61% less likely (AOR = 0.39; 95% CI: 0.28–0.52) to undergo TC compared to men from poor households.

## 4. Discussion

This study reported a TC prevalence of 51.0% (95% CI: 49.3–52.7) amongst men in Lesotho. This means that approximately half of the male population still practices TC, likely influenced by cultural or traditional beliefs. Findings from studies in other parts of Africa have also supported the idea that cultural reasons are a major factor in men choosing to undergo TC [38,39]. This research is crucial for Lesotho, a nation facing one of the highest global HIV rates and where TC is still frequently performed. The findings of combined multivariable multilevel logistic regression analysis showed that factors like age (35+ years), education level, literacy, smoking status, marital status (married), HIV test results, number of wives/partners, sexual history, residence area, district, and wealth status showed a significant association with TMC.

The findings also suggested that men aged 35 years and older were more likely to undergo TC compared to younger men (between 15 and 24 years). It was concluded that many young people do not value traditions and show little interest in cultural activities [40]. The findings indicated that men with a formal education were less likely to choose TC than those without any education. This may be because uneducated men are unaware of medical options and simply go through TC because they see no other alternatives. A study conducted in Tanzania found that many traditional circumcisers did not link TC to HIV as they believed the virus was mainly a problem found in urban areas [39]. Those men who could read just part of a sentence were 41% less likely to undergo TC compared to those who were completely illiterate. A similar reasoning to the one assigned above to men with no formal education may be attributed to the men who cannot read at all; they may not understand medical practices and the possible positive impact TC may have on preventing HIV transmission. This might be due to men who cannot read at all not fully understanding medical practices and the possible positive impact TC may have on preventing HIV transmission. One study found that most men undergo TC just for cultural reasons rather than HIV prevention [41]. Research conducted in South Africa revealed that 67 out of 100 men lacked awareness of the HIV transmission risks linked to TC, largely because of limited knowledge [42].

Men who smoked cigarettes, were married, and resided in rural areas were more likely to undergo TC than those in other groups. Previous studies suggested that men who underwent medical circumcision do not earn respect from the community compared to those who underwent TC [15,43]. This could be the reason that many married and rural men participating in this study underwent TC. Additionally, the study found that men living in the Berea and Maseru districts were less likely to undergo TC than those from the Butha-Buthe district. Similarly, men from households with middle to high incomes were less likely to undergo TC than those from lower-income backgrounds. A possible reason for this could be that men with greater financial resources could afford to access medical circumcision services.

TMC may affect the risk of HIV transmission through various mediating factors such as the timing of the procedure, the healing process of the circumcision wound, and the premature resumption of sexual activity. The timing of circumcision is a key factor in its effectiveness for reducing HIV risk. Circumcision performed after puberty (age 13 years and above) has been found to offer greater protection against HIV compared to circumcision carried out before puberty (age 12 years or younger) [44]. The period right after circumcision is crucial for proper wound healing. Research shows that the World Health Organization′s recommended 42-day abstinence period is ideal for both HIV-positive and HIV-negative men, as engaging in sexual activity before the wound has fully healed significantly raises the risk of HIV transmission. Early post-operative infections can further delay wound healing, exacerbating the risk [45].

A systematic review highlighted that traditionally circumcised men often believe that circumcision provides complete protection against HIV, which can lead to reduced condom use. One study cited in the review reported that 38% of traditionally circumcised men engaged in inconsistent condom use during sexual activity, while 8% reported never using condoms [46]. The finding of inconsistent condom use among traditionally circumcised men is further supported by a cohort study from the Eastern Cape Province, South Africa, which reported that 50% of these men used condoms inconsistently, and 19% never used condoms following circumcision [47]. A comprehensive meta-analysis conducted through November 2020 emphasized that while medical male circumcision significantly reduces HIV acquisition, risk compensation remains a concern. Several included studies reported decreased condom use and increased rates of multiple partnerships among circumcised men [48].

### Study Strengths and Limitations

This study used a national dataset, which allows for a thorough examination of factors associated with TMC amongst Lesotho men. In addition, the large sample size improves the accuracy of the estimates. However, the study has certain limitations. Its cross-sectional design captures information at only one point in time, which restricts the ability to infer causality and could lead to bias in cases where respondents complete the questionnaire whilst experiencing negative moods or emotions at that moment. Additionally, the literature review in this study included only articles published in English, possibly missing important information published in other languages. Furthermore, the researchers could not prove a direct causal link between the factors and TMC amongst men in Lesotho. Moreover, the study focused only on men and did not include their wives or partners to understand their perspectives and sexual behaviors associated with TMC.

## 5. Conclusions

Circumcision is closely related to public health, given that previous studies have shown it to lower the chances of HIV transmission. TMC is a cultural ritual performed away from home and is regarded as an important step in the journey from boyhood to manhood. This study’s findings suggest that TMC remains a common practice in Lesotho. Given the high percentage of men performing TC in communities, more research is needed on the risks of HIV transmission associated with unprotected sex following the procedure.

This research suggests that TC should be carried out by skilled and knowledgeable elders to ensure that the correct procedures are maintained, and participation should be voluntary for young people rather than mandatory. The authors recommend that future studies should focus on comparing the prevalence of performing TC amongst African countries since the sample for this study was limited to Lesotho. Furthermore, the researchers recommend that further studies compare the effectiveness of TC and medical circumcision in reducing HIV transmission, so that communities can be informed about the most effective methods, and so that appropriate public health interventions can be implemented.

## Figures and Tables

**Table 1:** Dataset description.

Variables	Definition and Coding
Is respondent traditionally circumcised	0 = No; 1 = Yes
Age group in years	1 = 15–24; 2 = 25–34; 3 = 35+
Place of residence	1 = Urban; 2 = Rural
District	1 = Butha-Bothe; 2 = Leribe; 3 = Berea; 4 = Maseru; 5 = Mafeteng; 6 = Mohale′s Hoek; 7 = Quthing; 8 = Qacha′s Nek; 9 = Mokhotlong; 10 = Thaba-Tseka
Level of education	0 = No education; 1 = Primary; 2 = Secondary; 3 = Higher
Sex of household head	1 = Male; 2 = Female
Literacy	0 = Cannot read at all; 1 = Able to read only parts of sentence; 2 = Able to read whole sentence; 3 = No card with required language
Wealth status	1 = Poorer; 2 = Middle; 3 = Richer
Smoke cigarette	0 = No; 1 = Yes
Covered by health insurance	0 = No; 1 = Yes
Marital status	0 = Never in union; 1 = Married; 2 = Living with partner; 3 = Windowed; 4 = Divorced; 5 = No longer living together/separated
Working	0 = No; 1 = Yes
HIV test result	1 = Positive; 2 = Negative; 3 = Indeterminate; 4 = Declined to answer
Would you marry a person with HIV	0 = No; 1 = Yes; 2 = Don’t know/not sure/depends
Number of wives/partners	0 = No wives/partners; 1 = one wife/partner
Self-reported health status	1 = Good; 2 = Moderate; 3 = Bad
Partner currently pregnant	0 = No; 1 = Yes; 2 = Unsure
Ever had sex	0 = No; 1 = Yes

**Table 2:** Characteristics of the weighted sample population for men reporting traditional circumcision (TC), 2023 LDHS (unweighted n  = 3208 and weighted n = 3202).

Variable		All Respondents, n (%)	TC	(*p*-Value)
			Yes (95% CI)	
Prevalence			51.0 (49.3–52.7)	
**Individual-level variables**				
Mean age (±SD) in years		32.4 (±12.3)	33.6 (±11.7)	
Age group (years)	15–24	1119 (35.0)	43.1 (40.2–46.0)	<0.001
	25–34	729 (22.8)	56.1 (52.5–59.7)	
	35 +	1354 (42.3)	54.8 (52.1–57.5)	
Level of education	No education	201 (6.3)	87.6 (83.0–92.2)	<0.001
	Primary	1192 (37.2)	71.9 (69.3–74.5)	
	Secondary	1375 (42.9)	38.9 (36.3–41.5)	
	Higher	434 (13.6)	15.0 (11.6–18.4)	
Literacy	Cannot read at all	381 (11.9)	82.1 (78.3–85.9)	<0.001
	Able to read only parts of sentence	436 (13.6)	64.4 (59.9–68.9)	
	Able to read whole sentence	2385 (74.5)	43.6 (41.6–45.6)	
Smoke cigarettes	No	1755 (54.8)	39.8 (37.5–42.1)	<0.001
	Yes	1447 (45.2)	64.6 (62.1–67.1)	
Covered by health insurance	No	2652 (82.8)	53.4 (51.5–55.3)	<0.001
	Yes	550 (17.2)	39.3 (35.2–43.4)	
Marital status	Never in union	1514 (47.3)	44.0 (41.5–46.5)	<0.001
	Married	1356 (42.3)	56.6 (54.0–59.2)	
	Living with partner	73 (2.3)	54.8 (43.4–66.2)	
	Widowed	68 (2.1)	64.7 (53.3–76.1)	
	Divorced	37 (1.1)	52.8 (36.7–68.9)	
	No longer living together/separated	154 (4.8)	62.3 (54.6–70.0)	
Working	No	1212 (37.9)	53.5 (50.7–56.3)	0.026
	Yes	1990 (62.1)	49.5 (47.3–51.7)	
Result of HIV test	Positive	507 (15.8)	58.4 (54.1–62.7)	<0.001
	Negative	2486 (77.6)	50.8 (48.8–52.8)	
	Indeterminate	99 (3.1)	41.4 (31.7–51.1)	
	Declined to answer	110 (3.4)	31.8 (23.1–40.5)	
Would you marry a person with HIV?	No	1373 (42.9)	53.6 (51.0–56.2)	<0.001
	Yes	1679 (52.4)	50.2 (47.8–52.6)	
	Don′t know/not sure/depends	151 (4.7)	36.4 (28.7–44.1)	
Number of wives/partners	No wives/partners	1773 (55.4)	46.6 (44.3–48.9)	<0.001
	One wife/partner	1429 (44.6)	56.5 (53.9–59.1)	
**Variable**		**All Respondents, n (%)**	**TC**	**(*p*-Value)**
			**Yes (95% CI)**	
Self-reported health status	Good	1672 (52.2)	48.4 (46.0–50.8)	<0.001
	Moderate	1160 (36.2)	51.0 (48.1–53.9)	
	Bad	370 (11.6)	63.0 (58.1–67.9)	
Partner currently pregnant	No	2069 (64.6)	53.6 (51.5–55.7)	<0.001
	Yes	906 (28.3)	43.8 (40.6–47.0)	
	Unsure	227 (7.1)	56.4 (49.9–62.9)	
Ever had sex	No	254 (7.9)	13.8 (9.6–18.0)	<0.001
	Yes	2949 (92.1)	54.2 (52.4–56.0)	
**Community-level variables**				
Place of residence	Urban	1319 (41.2)	34.7 (32.1–37.3)	<0.001
	Rural	1884 (58.8)	62.4 (60.2–64.6)	
District	Butha-Buthe	195 (6.1)	64.6 (57.9–71.3)	<0.001
	Leribe	624 (19.5)	58.7 (54.8–62.6)	
	Berea	477 (14.9)	40.4 (36.0–44.8)	
	Maseru	1012 (31.6)	35.1 (32.2–38.0)	
	Mafeteng	215 (6.7)	53.5 (46.8–60.2)	
	Mohale′s Hoek	161 (5.0)	62.1 (54.6–69.6)	
	Quthing	122 (3.8)	68.0 (59.7–76.3)	
	Qacha′s Nek	90 (2.8)	68.9 (59.3–78.5)	
	Mokhotlong	123 (3.8)	75.4 (67.8–83.0)	
	Thaba-Tseka	184 (5.8)	77.7 (71.7–83.7)	
Sex of household head	Male	2378 (74.3)	50.1 (48.1–52.1)	0.082
	Female	824 (25.7)	53.6 (50.2–57.0)	
Wealth status	Poorer	1127 (35.2)	75.2 (72.7–77.7)	<0.001
	Middle	705 (22.0)	55.5 (51.8–59.2)	
	Richer	1370 (42.8)	28.8 (26.4–31.2)	

CI = confidence interval; % = percentage; TC = traditional circumcision; n = sample size.

**Table 3:** Analysis outcomes of univariable and multivariable multilevel logistic regression for the determinants of TMC in Lesotho.

Variables	COR	Model 1	Model 2	Model 3	Model 4
	(95% CI)	(Null Model)	AOR (95% CI)	AOR (95% CI)	AOR (95% CI)
**Individual-level variables**					
**Age group**					
15–24	1		1		1
25–34	2.44 (1.94–3.07)		1.26 (0.95–1.67)	-	1.21 (0.90–1.61)
35+	2.51 (2.07–3.04)		1.61 (1.45–1.83)	-	1.63 * (1.46–1.86)
**Level of education**					
No education	1		1		1
Primary	0.30 (0.19–0.46)		0.49 (0.28–0.86)	-	0.54 * (0.30–0.97)
Secondary	0.08 (0.05–0.13)		0.14 (0.08–0.25)	-	0.17 * (0.09–0.32)
Higher	0.03 (0.02–0.04)		0.04 (0.02–0.07)	-	0.06 * (0.03–0.12)
**Literacy**					
Cannot read at all	1		1		1
Able to read only parts of sentence	0.36 (0.25–0.52)		0.56 (0.36–0.88)	-	0.59 * (0.37–0.93)
Able to read whole sentence	0.17 (0.13–0.24)		0.59 (0.39–0.90)	-	0.68 (0.44–1.03)
**Smoke cigarettes**					
No	1		1		1
Yes	3.33 (2.79–3.97)		1.99 (1.64–2.41)	-	1.82 * (1.50–2.21)
**Covered by health insurance**					
No	1		1		1
Yes	0.64 (0.49–0.83)		0.79 (0.60–1.03)	-	0.93 (0.70–1.22)
**Marital status**					
Never in union	1		1		1
Married	2.40 (2.00–2.88)		1.30 (0.98–1.72)	-	1.43 * (1.07–1.91)
Living with partner	2.79 (1.48–5.27)		1.40 (0.79–2.57)	-	1.77 (0.97–3.23)
Widowed	2.28 (1.29–4.03)		1.30 (0.62–2.71)	-	1.55 (0.73–3.32)
Divorced	3.25 (1.55–6.84)		1.92 (0.80–4.58)	-	2.32 (0.95–5.63)
No longer living together/separated	2.46 (1.62–3.74)		1.11 (0.69–1.78)	-	1.16 (0.72–1.86)
**Working**					
No	1		1		1
Yes	0.94 (0.79–1.13)		0.87 (0.70–1.06)	-	0.96 (0.78–1.18)
**Result of HIV test**					
Positive	1		1		1
Negative	0.69 (0.55–0.87)		0.90 (0.68–1.18)	-	0.88 (0.67–1.17)
Indeterminate	0.34 (0.21–0.55)		0.51 (0.28–0.93)	-	0.50 * (0.27–0.93)
Declined to answer	0.34 (0.21–0.57)		0.25 (0.14–0.45)	-	0.26 * (0.14–0.46)
**Would you marry a person with HIV?**					
No	1		1		1
Yes	1.05 (0.88–1.25)		0.99 (0.81–1.22)	-	1.04 (0.84–1.27)
Don’t know/not sure/depends	0.49 (0.32–0.75)		0.55 (0.35–0.88)	-	0.59 * (0.37–0.95)
**Number of wives/partners**					
No wives/partners	1		1		1
One wife/partner	2.11 (1.77–2.50)		2.24 (1.01–4.35)	-	3.12 * (1.71–5.13)
**Self-reported health status**					
Good	1		1		1
Moderate	1.22 (1.01–1.47)		1.11 (0.90–1.36)	-	1.14 (0.92–1.41)
Bad	1.68 (1.26–2.25)		0.97 (0.71–1.32)	-	0.93 (0.68–1.28)
**Partner currently pregnant**					
No	1		1		1
Yes	0.63 (0.52–0.76)		0.88 (0.69–1.13)	-	0.90 (0.70–1.15)
Unsure	0.81 (0.59–1.11)		0.95 (0.66–1.37)	-	0.95 (0.65–1.38)
**Ever had sex**					
No	1		1		1
Yes	13.37 (9.03–19.80)		10.91 (6.78–17.56)	-	12.42 * (7.69–20.07)
**Community-level variables**					
**Place of residence**					
Urban	1			1	1
Rural	4.66 (3.61–6.02)		-	1.52 (1.13–2.04)	1.54 * (1.12–2.12)
**District**					
Butha-Buthe	1			1	1
Leribe	0.66 (0.40–1.11)		-	0.90 (0.54–1.52)	0.73 (0.41–1.28)
Berea	0.34 (0.20–0.58)		-	0.46 (0.27–0.79)	0.39 * (0.22–0.70)
Maseru	0.21 (0.12–0.35)		-	0.40 (0.24–0.67)	0.33 * (0.19–0.57)
Mafeteng	0.58 (0.34–1.00)		-	0.69 (0.39–1.23)	0.65 (0.35–1.22)
Mohale’s Hoek	0.93 (0.53–1.63)		-	0.84 (0.45–1.55)	0.64 (0.33–1.25)
Quthing	1.21 (0.69–2.13)		-	0.84 (0.35–2.01)	0.83 (0.33–2.12)
Qacha’s Nek	1.27 (0.70–2.27)		-	1.12 (0.39–3.20)	0.87 (0.28–2.67)
Mokhotlong	1.72 (0.96–3.06)		-	1.18 (0.54–2.58)	0.99 (0.42–2.35)
Thaba-Tseka	1.91 (1.08–3.36)		-	1.06 (0.57–1.97)	0.74 (0.38–1.47)
**Sex of household head**					
Male	1			1	1
Female	0.86 (0.71–1.04)		-	0.90 (0.74–1.09)	1.24 (0.99–1.55)
**Wealth status**					
Poorer	1			1	1
Middle	0.41 (0.33–0.52)		-	0.57 (0.44–0.72)	0.72 * (0.55–0.95)
Richer	0.14 (0.11–0.18)		-	0.22 (0.17–0.29)	0.39 * (0.28–0.52)
**Random effect**					
Cluster variance		1.32	0.94	0.76	0.21
ICC		0.29	0.24	0.21	0.20
MOR		1.88	1.82	1.74	1.69
PCV		Reff	1.34	7.78	21.51
**Model comparison**					
Log-likelihood		−2011.701	−1985.521	−2001.632	−1986.202
AIC		4027.402	3851.922	4077.645	3804.416

Key: COR = crude odds ratio; AOR = adjusted odds ratio; 95% CI = 95 percent confidence interval; * = *p*-value < 0.05.

## Data Availability

All methods were carried out in accordance with relevant guidelines. and regulations, and data were sourced from the link below. The data supporting this article are available at: https://dhsprogram.com/data/dataset_admin/index.cfm (accessed on 26 November 2024).
